# Patterns of allergic rhinitis among adults in Qassim region, Saudi Arabia: a cross sectional study

**DOI:** 10.11604/pamj.2021.40.70.30719

**Published:** 2021-09-30

**Authors:** Sultan Alanazy, Mazyad Alenezi, Ibrahim Al-Quniabut, Ibrahim Al-juraifani, Malek Alburayh, Abdullah Altuaysi, Yahya Alyahya, Hajaj Al-Homaidan, Osama Al-Wutayd

**Affiliations:** 1 Department of Surgery, Ears Nose and Throat Unit, Unaizah College of Medicine and Medical Sciences, Qassim University, Al Qassim Saudi Arabia,; 2 Department of Otolaryngology, Head and Neck Surgery, College of Medicine, Qassim University, Al Qassim, Saudi Arabia,; 3 Unaizah College of Medicine and Medical Sciences, Qassim University, Al Qassim, Saudi Arabia,; 4 Department of Family and Community Medicine, Unaizah College of Medicine and Medical Sciences, Qassim University, Al Qassim, Saudi Arabia

**Keywords:** Allergic rhinitis, ENT, complications, presentation, triggers, Qassim, Saudi Arabia

## Abstract

**Introduction:**

developing and developed countries have a high prevalence of allergic rhinitis (AR). Severe AR has negative impacts on sleep, quality of life, and work performance. The study aimed to identify the patterns of AR among patients attending the ears nose and throat Unit (ENT) clinic at King Saud Hospital, Qassim, Saudi Arabia.

**Methods:**

this study was a cross-sectional study conducted at the ENT clinic of King Saudi Hospital, Unaizah City, Qassim region, Saudi Arabia. We examined outpatients diagnosed with AR using an interview questionnaire and clinical examination.

**Results:**

the sample included 455 patients. Of these, 23.7% were 21-30 years old, 65.7% had a family history of AR, 57.8% had no general symptoms, 75.6% reported runny nose as the most common nasal symptom, and 35.4% reported no complications. Dust was the most common trigger of AR (82.4%), 49.2% reported allergic symptoms in all seasons, 96% of patients have inferior turbinate hypertrophy, and oral histamine was the most commonly used treatment (33.2%).

**Conclusion:**

perineal AR and inferior turbinate hypertrophy were very common findings comparing to previous studies, further studies to assess the risk factors are highly recommended.

## Introduction

Rhinitis is a common inflammatory disorder of the nose, which affects approximately 40% of the population [1]. There are several different types of rhinitis, but allergic rhinitis (AR) is the most common type, reported to affect approximately 10%-20% of the population [2]. Allergic rhinitis prevalence is high in both developing and developed countries [3], affecting almost 400 million individuals worldwide [4,5]. In Jazan City in Saudi Arabia, an AR incidence of approximately 44% has been reported, substantially exceeding the global average of 10%-20% [6]. Symptoms of AR typically emerge after exposure to an allergen due to IgE mediated inflammation of the membranes lining the nose [7]. The symptoms of AR result in fatigue, cognitive function decline which impairs quality of life, depressed mood and sleep disturbances [8]. There are several triggers for AR, including domestic animals, mites, allergens of plant origin or animal origin, common outdoor allergens such as mold and pollens, tobacco smoke, occupational triggers such as latex, sulfur oxide and oxides of nitrogen, aspirin, ozone, and other non-steroid anti-inflammatory medications [7]. The major symptoms of AR include nasal obstruction, itching postnasal drip, sneezing and rhinorrhea [9]. AR can be classified into seasonal or perennial the former occurs during a specific season, whereas the later occurs throughout the year [10].

However, because this classification is not able to describe all patients, another classification system was established in which AR is classified according to symptom duration, as either intermittent (inflammation duration of less than 6 months) or persistent (symptoms continue throughout the year) and severity is classified as mild (where the patient can sleep normally), moderate or severe (where sleep and daily activity are affected) [11,12]. Because AR is commonly undetected in primary care settings, with patients suffering from the condition often being unable to recognize the impact of the disease and physicians failing to regularly ask patients about the condition, screening for AR is recommended [13]. Saudi Arabia is known for its frequent and periodic sandstorms in all seasons. Sandstorms transport many types of microorganisms and dust particles that can trigger or exacerbate respiratory diseases such as AR and asthma [14]. Qassim region is a central area in the kingdom of Saudi Arabia, and is well known agricultural zone, and has the desert climate, with frequent dust storms, and low humidity [15]. In unpublished communication, medical practitioners in various specialties have thought that a large number of people in Qassim region suffer from AR compared to other regions. However, few studies of this widespread disease have been conducted in Saudi Arabia, and the patterns of the disease remain to be clarified. To the best of our knowledge, this is the first study of AR to be conducted in Qassim region. The present study was conducted to identify patterns of AR among patients attending the ear, nose and throat (ENT) outpatient clinic at King Saud Hospital, Unaizah City, Qassim region, Saudi Arabia.

## Methods

**Study design and setting:** we conducted a quantitative observational descriptive cross-sectional study among outpatients from the ENT clinic of King Saud Hospital, which has a capacity of 294 beds and is the only governmental hospital in Unaizah city, Qassim region, between January 2019, and December 2019.

**Sample size:** the sample size was calculated based on assuming that the prevalence of perineal allergic rhinitis was 50% to get the maximium sample size, type I error 5%, adequate power (80%), and 15% of expected incomplete data. The minimium sample size was 443.

**Sampling, data collection and analysis:** a systematic sampling technique was used to enroll participants over 18 years of age. Patient's who were under 18 years of age were excluded. An interview questionnaire was used to collect data, and was given to the doctors to fill in during the interview with patients. The questionnaire focused on personal data, clinical presentation, recurrent triggers, family history, and co-morbidities according to the study objective and based on previous studies [6,16-18]. Clinical examinations were performed, including a detailed ENT examination using an endoscope. The diagnosis of AR was based on clinical diagnosis when patients have a history, and physical findings which compatible with allergic cause according to American academy of otolaryngology head and neck surgery [19]. The data were entered into excel then transferred into STATA v16 for analysis. Description of data was conducted using frequency and percentages for categorical variables.

**Ethical considerations:** ethical approval was obtained from the regional committee of bioethics in Qassim region before we began the process of data collection. Written consent was obtained from all participants before participation in the study.

## Results

The response rate was 97%, and 455 participants were recruited to the study. The most common age group was 21-30 years old (n= 108 23.7%). There were more male than female participants (n=286 62.9% vs n= 169 37.1%, respectively). The most common body mass index category was normal weight (n=203 44.6%). The sample contained 53 (11.6%) office workers, and most participants lived in the city (n= 420 72.3%). The most commonly reported type of home was villa (n=198 43.5%). More than three quarters of participants were non-smokers (n= 363 79.8%), whereas there were 44 (9.7%) smokers. Of the 44 smokers, 24 (54.5%) participants reported smoking for more than 10 years, and 25 (65.8%) participants reported smoking ≤ 1 pack per day. Most participants (412 90.5%) reported that they did not undergo an allergy test, while 43 (9.5%) participants had undergone an allergy test. Of the 43 participants who underwent the allergy test, 15 (34.9%) participants reported that they underwent an allergy test more than 5 years earlier, and the skin test was the most common type (n= 36 83.7%). More than half of the participants reported having a family history of allergy (n= 299 65.7%), as shown in Table 1. The clinical presentations of participants are shown in Table 2. More than half of participants (n= 263 57.8%) reported no general symptoms, while 188 (41.3%) and 68 (14.9%) reported fatigue and malaise, respectively. Commonly, the onset of symptoms was ≤10 years (n= 216 47.5%). The most common nasal, throat and sinus symptoms were runny nose 344 (75.6%), frequent clearing 205 (45.1%) and headache 215 (47.3%), respectively. Among the 215 participants who reported headache characteristics, the most commonly reported location of headache was frontal (n= 106 49.3%). Most participants experiencing headache (n= 20595.3%) reported tension as the type of headache, and more than half of them (n= 124 57.7%) reported that it occurred occasionally.

**Table 1:** number and percentage of descriptive data of the studied participants (n=455)

Characteristics	Number	%
Age:		
< 20	99	21.8
21-30	108	23.7
31-40	90	19.8
41-50	70	15.4
51-60	56	12.3
> 60	32	7
**BMI**		
Underweight	23	5
Normal weight	203	44.6
Overweight	101	22.2
Obese	128	28
**Gender**		
Male	286	62.9
Female	169	37.1
**Occupation**		
Child	49	10.8
Teacher	35	7.7
Office worker	53	11.6
Outdoor worker	34	7.5
Other	284	62.4
**Living in:**		
City	420	92.3
Village	35	7.7
**Type of home**		
Villa	198	43.5
Apartment	81	17.8
Farm	8	1.8
Other	168	36.9
**Smoking:**		
Smoker	44	9.7
Passive smoking	48	10.5
Non-smoker	363	79.8
**if yes: for how many years? (n=44**)		
≤ 10 years	20	45.5
> 10 years	24	54.5
**How many packs per day? (n=44)**		
≤ 1 Pack	25	56.8
> 1 pack	19	19 (43.2)
**Allergy test yes/no**		
Yes	43	9.5
No	412	90.5
**If yes, then the time from the last test is: (n=43**)		
< 1 year	12	27.9
1-2 years	13	30.2
2-5 years	3	7
> 5 years	15	34.9
**Type of test used: (n=43)**		
Skin	36	83.7
Blood	7	16.3
**Family history**		
Yes	299	65.7
No	156	34.3

**Table 2:** number and percentage of clinical presentation of the studied participants

Clinical presentation	Number	%
**General symptoms**		
Fatigue	188	41.3
Malaise	68	14.9
No general symptoms	263	57.8
**Onset of symptoms**		
≤ 10 years	216	47.5
11-30 years	127	27.9
> 30 years	112	24.6
**Onset of symptoms**		
≤ 5 years	145	31.9
6-10 years	71	15.6
11-15 years	16	3.5
16-20 years	42	9.2
> 20 years	181	39.8
**Nasal symptoms**		
Frequent sneezing	336	73.8
Runny nose	344	75.6
Nasal congestion	343	75.4
Nasal itching	254	55.8
Frequent nose bleeds	80	17.6
Loss of smell	160	35.2
**Throat symptoms**		
Frequent clearing	205	45.1
Palate itching	170	37.4
Sore throat (pain)	63	13.8
No throat symptoms	150	33
**Sinus symptoms**		
Pressure on cheeks	91	20
Pressure around eyes	171	37.6
Post-nasal drip	323	71
Headache	215	47.3
No sinus symptoms	166	36.5
**Location of headache (n=215)**		
Frontal	106	49.3
Temporal	30	14
Back of head	3	1.4
Sinus	31	14.4
Whole head	46	21.4
**Type of headache (n=215)**		
Tension	205	95.3
Migraine	10	4.7
**Frequency of headache (n=215)**		
Daily	27	12.6
Occasionally	124	57.7
Seldom	64	29.8
**Skin symptoms**		
Itching	115	25.3
Eczema	47	10.3
Contact rash	41	9
No skin symptoms	325	71.4
**Lung symptoms**		
Asthma	130	28.6
Wheezing	37	8.1
Coughing	102	22.4
Worsening with exercise	31	6.8
No lung symptoms	269	59.1
**Eye symptoms**		
Itching	232	51
Redness	237	52.1
Watery discharge	231	50.8
Burning	126	27.7
No eye symptoms	128	28.1
**Ear symptoms**		
Fullness	95	20.9
Pain	56	12.3
Decreased hearing	59	13
Ear itching	143	31.4
No ear symptoms	244	53.6

Most participants reported no skin symptoms (n= 325 71.4%), more than half (n= 269 59.1%) reported no lung symptoms, and 244 (53.6%) reported no ear symptoms, whereas the most commonly reported eye symptom was redness of the eye (n= 237 52.1%). The results revealed that 161 (35.4%) participants reported no complications, whereas the most commonly reported complications were sleep disturbance, acute or chronic sinusitis, dental problems, and otitis media (n= 126 27.7%, n= 85 18.7%, n= 69 15.2% and n= 9 2%, respectively. The majority of patients reported mild symptoms (n= 368 80.9%), and 340 (74.7%) participants reported that symptoms were intermittent. Regarding the situations in which patients experienced their symptoms, the most common response was any place (n= 397 87.3%). Regarding triggers of allergic rhinitis, dust (n= 375 82.4%), perfume (n= 264 58%), and cold weather (n = 17939.3%) were the most common triggers, while 224 (49.2%) participants reported that their symptoms occur in all seasons of the year. There were 314 (69%) participants who reported that they avoid certain triggers to control their symptoms, as shown in Table 3. Patients´ examination results are shown in Table 4. Regarding the nose, 436 (96%) participants had hypertrophy inferior turbinate, while 345 (76%) participants had pale bluish discoloration of the nasal mucosa. Eye examination results revealed that 67 (14.7%) participants had watery discharge. Throat and pharynx examination revealed that 227 (50%) participants had post nasal drip. Regarding ear examination, 109 (24%) participants had tympanic membrane retraction and three (0.7%) participants had otitis media with effusion. Examination of nasopharynx revealed that 32 (7%) participants had hypertrophy mucosa and nine (2%) participants had discharge. Moreover, seven (1.5%) and four (0.9%) participants exhibited allergic shiner and salute, respectively, as additional findings. Regarding the treatments used for allergic rhinitis, 166 (36.5%) participants used no medication, 151 (33.2%) used oral histamine, and 141 (31%) used intranasal steroid

**Table 3:** number and percentage of description of allergic rhinitis of the studied participants

Description	Number	percent(%)
**In what situation does the patient experience symptoms?**		
At home	14	3.1
At work	9	2
At parks	12	2.6
Any place	397	87.3
Other	23	5.1
**Triggers**		
Smoke	160	35.2
Dust	375	82.4
Perfume	264	58
Cold weather	179	39.3
Pets	112	24.6
Air conditioning	151	33.2
Other	20	4.4
**Seasons**		
Summer	159	34.9
Winter	83	18.2
Autumn	9	2
Spring	22	4.8
All seasons	224	49.2
Avoidance of triggers to control symptoms	314	69

**Table 4:** number and percentage of clinical examination of the studied participants

Clinical examination	Number	percent(%)
**Nasal**		
Nasal discharge	309	68
Hypertrophy inferior turbinate	436	96
Pale bluish discoloration of the nasal mucosa	345	76
Nasal septal deviation	166	36.5
Nasal polyps	38	8.4
Other	16	3.5
**Eye**		
Watery discharge	67	14.7
Swollen conjunctiva (cobblestone)	18	4
Scleral injection (red eyes)	38	8.4
Periorbital puffiness	29	6.4
Others	6	1.3
**Throat and pharynx**		
Post nasal drip	227	50
Prominent (cobblestone) pharyngeal mucosa	36	8
**Ears**		
Otitis media with effusion	3	0.7
Tympanic membrane retraction	109	24
**Nasopharynx**		
Hypertrophy mucosa	32	7
Discharge	9	2
**Other findings**		
Allergic shiner	7	1.5
Allergic salute	4	0.9

## Discussion

Clinically, AR is defined as a symptomatic nose disease evoked by IgE-mediated inflammation of the nasal membrane after exposure to an allergen [20]. It is the most common respiratory disease, affecting 19% and 8.8%-16% of the general population in Europe and the United States, respectively [21,22]. AR affects both young adults and teenagers frequently. However, the prevalence of AR has been found to decrease after the age of 20 years old [23]. A previous study from Jazan region in Saudi Arabia reported that the prevalence of AR was 44% among adults [6]. In the present study, the findings indicate that AR prevalence peaked at between 22-31 years old, and decreased with increasing age. A study with a similar finding in Jazan city reported that, the peak of age of AR was 22 years old, which constitutes 25.8% [6]. Also in our study, 62.9% was male and 37.1% was female which is inconsistence with study conducted in Saudi Arabia using electronic survey found more females with AR than males, however this is may be due to selection bias [24]. It was previously reported that having a parent with AR increased the risk of having AR [25], while other reported risk factors include exposure to cigarette smoke, obesity, increased blood eosinophils, amplified IgE in the serum, and environmental factors such as environmental exposure in urban areas [26,27]. In contrast, growing up in a farming environment was associated with reduced risk of AR [28]. In the current study, 65.7% of patients reported having a family history of AR. This consistent with study conducted in Saudi Arabia, in which most of AR patient have positive family history (64.1%) [24].

Regarding obesity, half of participants were overweight and obese. The percentage of normal weight is consistent with study conducted in Saudi Arabia, in which AR occurred in normal weight individual constituting 38%, while overweight and obesity constitute 58% [24]. A previous study reported association between the high body mass index and AR [29], while another study reported no association [30], suggesting that this issue may require further investigation. Moreover, 79.8% of our patients reported that they were not smokers, 9.7 % of them are smoker and 10.5 % are passive smoker indicating that smoking was not a risk factor for this study group. This consistence with study done in Al-Ahssa in which 6% of patients are smokers and 8% are passive smokers [31], but in contrast to a study done in western area of Saudi Arabia concluded that 84.8% are either smokers or ex-smokers and only 15.2% non-smoker [16]. Regarding residence area, 92.3% of participants were living in the city which is similar to the study of western area of Saudi Arabia reported that 91% are urban citizen [16].

The majority of patients reported mild symptoms 80.9%), and (74.7%) participants reported that symptoms were intermittent. This inconsistent with study done in Riyadh, where 34% of AR reporting mild symptoms and 54% reporting intermittent symptoms [24]. Skin symptoms, lung symptoms, and ear symptoms were uncommon in this study, and more than half of the participants had no general symptoms. The most common reported nasal symptoms were runny nose (75.6%), nasal obstruction (75.5%) and frequent sneezing (73.8%). This is consistence with a study where the most common present symptoms were runny nose (82%), nasal itching (70%) and nasal obstruction (69%) [32], also in the study survey conducted an Middle East in five countries Saudi Arabia one of them, the percentage of symptoms reported by the participants were runny nose (57%), nasal itching (56%), nasal congestion (55%), throat itching (52%), reduced sense of smell (51%), and postnasal drip (50%) [17]. Complications of AR included chronic and acute sinusitis, apnea or sleep disturbance, dental problems and otitis media [18]. The current results revealed that 35.4% of patients had no complications, while the most common complications were sleep disturbance, acute or chronic rhinitis and dental problems, whereas otitis media represented only 2% of cases among all complications. In present study, sleep disturbance present in 27.7% of cases with AR. In the study done in Al-Ahssa, sleep disturbance were reported in 75% of cases with AR [31], and in the study survey conducted in Middle East in five countries Saudi Arabia one of them, the percentage of sleep disturbance were reported by about 80% [31]. The goal of AR treatment is to relieve symptoms, and there are several therapeutic options, including oral histamines, leukotriene, allergen immunotherapy, avoidance measures and intra-nasal corticosteroids [13]. In the current study, the most common treatments were oral histamine (33.2%), intranasal steroids (31%), and nasal decongestant. Although the study was selecting a representative sample from the study population, however, there are some limitations that should be considered. First, the study design was descriptive cross sectional that providing a snapshot of the frequency of each variable and did not asses the relationship between exposure and outcome. Second, recall bias cannot be rule out as the nature of the study design.

## Conclusion

Allergic rhinitis decreases with increasing age. Most patients with AR were living in urban areas and had a family history of AR. Thus, these characteristics appear to constitute risk factors for AR. The most common symptoms of AR were non-specific and mild, and nasal symptoms were the most frequent. Perineal allergic rhinitis was the common type and inferior turbinate hypertrophy with pale bluish discoloration was the most common finding during the clinical examination. Dust was the most common trigger for AR. Oral histamine and intranasal steroids were the most commonly used treatments.

### What is known about this topic

- Allergic rhinitis has wide varities of presentations and complications;
- Allergic rhinitis had negative impact on quality of life;
- Allergic rhinitis management are avoidance, medications and immunotherapy.

### What this study adds

- This is the first study to assess common presentations and patterns of allergic rhinitis in Qassim region;
- Significantly, the perineal allergi rhinitis was the most common frequent type;
- Interestingly, the inferior turbinate hypertrophy was common finding in physical examination.
